# Age-Related Decrease in Interthalamic Adhesion Thickness in Cats Without Apparent Cognitive Dysfunction

**DOI:** 10.3390/ani16203317

**Published:** 2026-10-21

**Authors:** Réjane Kerbrat-Copy, Océane Thérier, Marion Fusellier

**Affiliations:** 1 Department of Morphology, Imaging, Orthopedics, Rehabilitation, and Nutrition, Faculty of Veterinary Medicine, Ghent University, 9820 Merelbeke, Belgium; 2 Small Animal Diagnostic Imaging Department, Veterinary Hospital, Oniris College of Veterinary Medicine Food Science and Engineering, 44300 Nantes, Francemarion.fusellier@oniris-nantes.fr (M.F.); 3 Regenerative Medicine and Skeleton, RMeS, UMR 1229, INSERM, Oniris, Faculty of Dentistry, Nantes Université, 44036 Nantes, France

**Keywords:** interthalamic adhesion, cerebral atrophy, cognitive dysfunction syndrome, feline, aging

## Abstract

Feline cognitive dysfunction syndrome (FCDS) is an age-associated disorder for which objective imaging markers remain limited. This study evaluated the association between age and interthalamic adhesion thickness (IAT) in cats without apparent cognitive dysfunction and described IAT across feline life stages. MRI examinations from 112 client-owned cats aged 4 months to 18 years were retrospectively reviewed. Cats were classified as kittens (<1 year), young (1 to < 7 years), mature (7 to <11 years) and senior (≥11 years). Cats with intracranial lesions or reported behavioral changes suggestive of cognitive dysfunction were excluded. Two blinded observers measured IAT on transverse T1W and T2W images. Analyses included linear regression adjusted for brain height, brain width, sex/neuter status, breed group, and scanner; exploratory quadratic age modeling; and comparisons among life stages. Interobserver agreement was high. Age was negatively associated with IAT on T1W (B = −0.071 mm/year) and T2W images (B = −0.067 mm/year; both *p* < 0.001). This association persisted after multivariable adjustment (T1W: B = −0.074 mm/year; T2W: B = −0.067 mm/year; both *p* < 0.001) and after excluding kittens or brachycephalic cats. Senior cats had lower IAT than all other life stages on both sequences. A quadratic term suggested a steeper decline at older ages on T1W images only; this finding remains exploratory. These findings support a group-level association between age and IAT, rather than individual-level diagnostic use. Its potential diagnostic value for FCDS requires prospective evaluation with standardized behavioral assessment and age-matched controls.

## Simple Summary

Feline cognitive dysfunction syndrome is an age-related condition that can cause changes in behavior. It is difficult to diagnose because the clinical signs may be subtle and can also occur with other medical conditions. Brain magnetic resonance imaging may help identify objective changes associated with brain aging, but normal reference information in cats is limited. This study examined the interthalamic adhesion, which is a small cerebral structure already studied for cognitive dysfunction in dogs and humans. Brain scans from 112 cats aged 4 months to 18 years without reported signs of cognitive dysfunction were reviewed. The thickness of this structure decreased with age and was particularly smaller in the oldest age groups. This association persisted after accounting for brain dimensions, sex and neuter status, breed group, and MRI scanner, and after excluding kittens or cats of brachycephalic breeds. These findings provide additional information on age-associated brain measurements in cats undergoing clinical imaging. However, they do not establish whether this measurement can distinguish aging without cognitive dysfunction from feline cognitive dysfunction syndrome. Further studies comparing cats with and without confirmed cognitive dysfunction, using standardized behavioral assessments and imaging protocols, are needed before its diagnostic value can be determined.

## 1. Introduction

Advances in veterinary care have contributed to increased life expectancy in domestic cats, resulting in a higher prevalence of age-related diseases, including feline cognitive dysfunction syndrome (FCDS). The prevalence of this condition increases with age, and its clinical signs are frequently interpreted by owners as part of normal aging. FCDS is characterized by an age-associated decline in cognitive function that manifests as behavioral changes not attributable to other medical conditions [1], such as pain, endocrinopathies (e.g., hyperthyroidism), and diseases affecting the central nervous system [2]. A previous review reported that owners observed at least one behavioral change in 36% of cats aged 7–11 years, 28% of cats aged 11–14 years, and 50% of cats older than 15 years; the reported prevalence reached 88% in cats aged 16–19 years [3].

Despite its clinical relevance, limited data are available regarding the diagnosis of FCDS, its risk factors, and its association with brain atrophy [4]. Brain atrophy has been documented during normal aging in cats, consistent with findings in dogs and humans. However, the relationship between FCDS and brain atrophy remains insufficiently investigated, although it is suspected to play a significant role [5].

In dogs, cognitive dysfunction syndrome has been investigated more extensively, and similar diagnostic challenges have prompted the evaluation of objective imaging markers of brain atrophy. Among these markers, the dorsoventral interthalamic adhesion thickness (IAT) has emerged as a potentially useful parameter. The interthalamic adhesion is a rounded midline bridge of gray matter connecting the right and left thalami across the third ventricle. In dogs, interthalamic adhesion size has been negatively correlated with age in healthy individuals [6,7], and has been reported to be lower in dogs with cognitive dysfunction [7,8,9], supporting its use as an indicator of cerebral atrophy.

In cats, however, age-related changes in interthalamic adhesion size remain unclear. Published studies have yielded conflicting results. A study using voxel-based morphometry did not identify a significant decrease in interthalamic adhesion size in healthy cats [5], although this finding should be interpreted cautiously. This voxel-based morphometry analysis was not designed as a dedicated assessment of the interthalamic adhesion, and the authors noted that subtle changes in small localized regions of the feline brain may not be detectable with this approach. Conversely, another study reported significantly lower IAT in older cats (≥12 years); however, this finding was based on a small sample distributed across only three age categories [10]. Additionally, a recent study that evaluated age as a continuous variable also reported a significant decrease in interthalamic adhesion area and in the ratio of IAT to brain height with increasing age [11]. However, that study also included a small sample of cats.

Therefore, this study primarily aimed to evaluate the association between age, modeled as a continuous variable, and IAT in cats without apparent cognitive dysfunction, and secondarily to describe IAT across feline life stages. Establishing such baseline values is essential for future comparisons between healthy cats and age-matched cats exhibiting clinical signs consistent with FCDS. The authors hypothesized that IAT would significantly decrease with increasing age.

## 2. Materials and Methods

### 2.1. Study Design and Case Selection

This retrospective cross-sectional study was conducted at the Small Animal Diagnostic Imaging Department of the Veterinary Hospital, Oniris (Nantes, France). Client-owned cats of any age that underwent clinically indicated brain or skull MRI between 2007 and 2023 were eligible for inclusion. Cats were excluded if MRI revealed an apparent cerebral parenchymal lesion, such as a mass lesion, encephalitis or hydrocephalus, or if the owner reported behavioral changes suggestive of cognitive dysfunction. For each cat, age, body weight, sex, breed, MRI indications, and final diagnosis were recorded.

The cats were classified into four age categories, as described in the most recent AAHA/AAFP Feline Life Stage Guidelines [12]: kitten (<1 year), young adult (1 to <7 years), mature adult (7 to <11 years) and senior (≥11 years).

### 2.2. MRI Procedure

MRI examinations performed between 2007 and August 2015 were acquired with a 1.0 Tesla scanner (Magnetom Harmony, Siemens Medical Solutions, Erlangen, Germany), whereas examinations performed between September 2015 and 2023 were acquired with a 1.5 Tesla scanner (Magnetom Essenza, Siemens Medical Solutions, Erlangen, Germany) equipped with a standard head coil. In all examinations, transverse T1-weighted (T1W) and T2-weighted (T2W) sequences were acquired.

On the 1.0 T system, the image matrix was 384 × 228 pixels, with a DICOM pixel spacing of 0.469 × 0.469 mm/pixel, corresponding to an in-plane spatial resolution of approximately 0.47 mm/pixel. The acquisition matrix was 384 × 228. Slice thickness was 3.0 mm, with a 3.0 mm spacing between successive slices, corresponding to contiguous slices without an interslice gap. The percent sampling was 100%, and the percent phase field of view was 60%. T1W images were obtained using a repetition time (TR) of 575 ms and an echo time (TE) of 12 ms. T2W images were acquired using a TR of 4300 ms and a TE of 95 ms.

On the 1.5 T system, the reconstructed image matrix was 448 × 448 pixels, with a DICOM pixel spacing of 0.379 × 0.379 mm/pixel, corresponding to an in-plane spatial resolution of approximately 0.38 mm/pixel. The acquisition matrix was 448 × 359. Slice thickness was 3.0 mm, with a 3.3 mm spacing between successive slices, corresponding to a 0.3 mm interslice gap. The percent sampling was 80%, and the percent phase field of view was 100%. T1W images were obtained with a TR of 475 ms and a TE of 12 ms. T2W images were acquired with a TR of 4850 ms and a TE of 94 ms.

MRI examinations were performed with cats under general anesthesia and positioned in dorsal recumbency for all image acquisitions.

### 2.3. Interthalamic Adhesion Thickness, Brain Width and Brain Height Measurements

**Figure 1:**
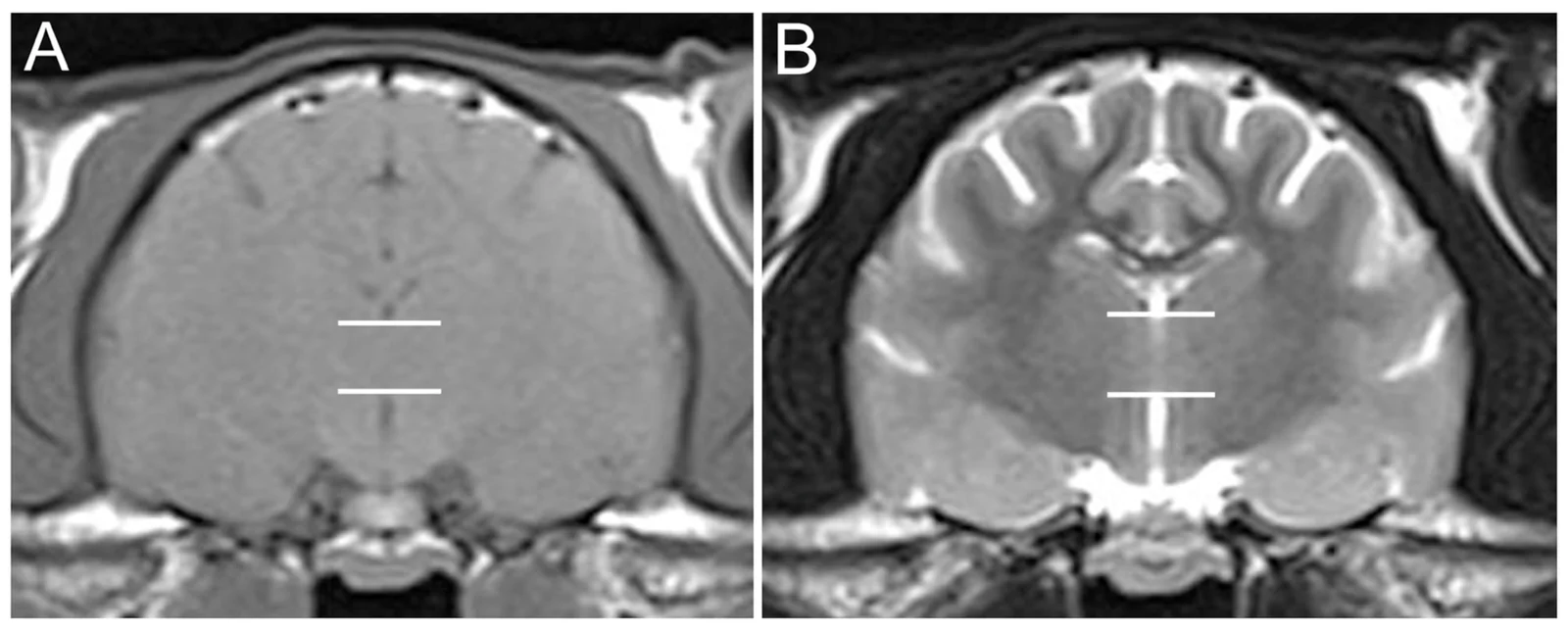
Measurement of the interthalamic adhesion thickness (between white lines) on transverse MR images, on a T1W sequence (**A**) and a T2W sequence (**B**).

IAT measurements were performed using Horos software (version 4.0.1; Horos Project) by two independent observers: a professor of diagnostic imaging and a final-year veterinary student who had completed specific training. The observers were blinded to age category and all other clinical information. IAT was measured on transverse T1W and T2W MR images of the brain at the level of its maximal dorsoventral dimension (Figure 1). Brain height and width were measured for each cat by the final-year veterinary student on the same transverse T1W image used for IAT measurement, as the dorsoventral distance between the dorsal and ventral margins of the brain parenchyma and the transverse distance between its right and left lateral margins, respectively, to obtain global brain dimensions for subsequent morphometric analyses and to evaluate potential associations between brain size and IAT.

### 2.4. Statistical Analysis

Statistical analyses were performed using R (version 4.6.0; R Foundation for Statistical Computing, Vienna, Austria) within RStudio (version 2026.09.0+174; Posit Software, PBC, Boston, MA, USA).

#### 2.4.1. Assessment of Measurement Reproducibility

Reproducibility of the measurement protocol was assessed by evaluating interobserver agreement for IAT measurements independently obtained by two operators from the same T1W and T2W MR images.

Agreement was assessed using Bland–Altman analysis, the Lin concordance correlation coefficient, and a two-way mixed-effects correlation coefficient (ICC) for absolute agreement based on the mean of the two observers’ measurements [ICC(3,k)]. Bland–Altman plots were used to evaluate agreement by plotting the difference between the two observers’ measurements against their mean and to estimate mean bias and 95% limits of agreement. The Lin concordance correlation coefficient (ρc), together with its accuracy component (Cb) and precision component (Pearson’s correlation coefficient, ρ), was calculated to quantify agreement. ICC values and their 95% confidence intervals (CIs) were calculated to summarize interobserver agreement.

The standard error of measurement (SEM) and minimal detectable change at the 95% confidence level (MDC95) were estimated separately for each scanner and sequence using paired measurements from the two observers. Assuming independent random measurement errors of equal variance, SEM was calculated as the standard deviation of the differences between observers divided by $\sqrt{2}$. MDC95 was calculated as 1.96 × $\sqrt{2}$ × SEM.

#### 2.4.2. Associations Between IAT, Age and Morphometric Variables

For each cat, IAT values used in subsequent analyses were calculated as the mean of the two observers’ measurements for T1W and T2W images separately.

Associations between T1W IAT and brain width, brain height and body weight were assessed in separate linear regression models, each adjusted for age and scanner. Age was included to distinguish associations with these morphometric variables from the age-related variation in IAT, while scanner was included to account for differences between the two MRI systems.

Age was modeled as a continuous variable (exact age in years at the time of MRI). The association between age and IAT was assessed with simple linear regression, separately for T1W and T2W images. Multivariable linear regression models, including age, brain height, brain width, sex/neuter status, breed group (brachycephalic or non-brachycephalic) and scanner, were fitted separately for T1W and T2W measurements. Collinearity was assessed using adjusted generalized variance inflation factors.

To explore a possible acceleration of IAT decline with age, a quadratic term (age^2^) was added to the linear model, and the two models were compared using Akaike’s information criterion (AIC) and an F-test for nested models. Model fit was summarized with R^2^, and the assumptions of linearity, normality and homoscedasticity of residuals were assessed visually with residual plots and Q–Q plots.

Sensitivity analyses were performed by repeating the age analyses after exclusion of kittens (<1 year). Additional sensitivity analyses assessed the linear association between age and IAT after exclusion of brachycephalic cats (Persian and Exotic Shorthair breeds), separately for T1W and T2W measurements.

#### 2.4.3. Comparisons Among Age Categories

To allow comparison between life stages, mean IAT was also compared among age categories: kitten, young, mature and senior. The association between age category and IAT was assessed with a linear model in which age category was included as a categorical explanatory variable. Normality of model residuals was assessed by visual inspection of Q–Q plots. A one-way analysis of variance (ANOVA) was performed to assess whether mean IAT differed among age groups. Statistical significance was set at *p* < 0.05. When a significant effect of age was detected, pairwise comparisons among age groups were performed using Tukey’s post hoc test.

## 3. Results

### 3.1. Study Population

A total of 112 cats aged 4 months to 18 years were included (9 kittens, 56 young, 24 mature and 23 senior). Age, body weight, sex, breed distribution and brain width and height for each group are summarized in Table 1.

The median age was 5.56 years in cats examined using the 1.0 T scanner (*n* = 29; 25th–75th percentiles, 1.51–8.06 years; range, 0.31–14.50 years) and 5.46 years in cats examined using the 1.5 T scanner (*n* = 83; 25th–75th percentiles, 2.79–10.20 years; range, 0.35–17.96 years).

No included cats had apparent cerebral parenchymal lesions on MRI, including mass lesions, encephalitis and hydrocephalus. Recorded diagnoses included idiopathic epilepsy, ear disease (external, middle or internal ear), nasal disease, nasopharyngeal polyps, craniocervical junction malformations, cervical spinal cord disease, intoxication or no abnormalities identified.

### 3.2. Measurement Reproducibility

For T1W images, Bland–Altman analysis showed a mean bias of −0.08 mm, with 95% limits of agreement ranging from −0.68 to 0.53 mm. The Lin concordance correlation coefficient was ρc = 0.87 (0.82–0.91), precision ρ = 0.88, and accuracy Cb = 0.99. The ICC was 0.93 (95% CI: 0.90–0.95), indicating excellent interobserver agreement. Overall, acceptable agreement was observed between observers.

For T2W images, Bland–Altman analysis showed a mean bias of −0.07 mm, with 95% limits of agreement ranging from −0.62 to 0.47 mm. The Lin concordance correlation coefficient was ρc = 0.89 (0.85–0.92), precision ρ = 0.90, and accuracy Cb = 0.99. The ICC was 0.94 (95% CI: 0.92–0.96), indicating excellent interobserver agreement. Overall, acceptable agreement was observed between observers.

Scanner-specific estimates of the interobserver SEM ranged from 0.179 to 0.238 mm, and the corresponding MDC95 estimates ranged from 0.497 to 0.658 mm. On the 1.0 T images (*n* = 29), SEM and MDC95 were 0.188 and 0.520 mm for T1W measurements and 0.238 and 0.658 mm for T2W measurements, respectively. On the 1.5 T images (*n* = 83), the corresponding values were 0.228 and 0.633 mm for T1W measurements and 0.179 and 0.497 mm for T2W measurements.

### 3.3. Associations Between IAT, Age and Morphometric Variables

In separate linear regression models adjusted for age and scanner, T1W IAT was not significantly associated with brain width (B = 0.032 mm/mm, 95% CI −0.011 to 0.074, *p* = 0.142), brain height (B = 0.045 mm/mm, 95% CI −0.002 to 0.092, *p* = 0.059), or body weight (B = 0.060 mm/kg, 95% CI −0.011 to 0.131, *p* = 0.097).

**Figure 2:**
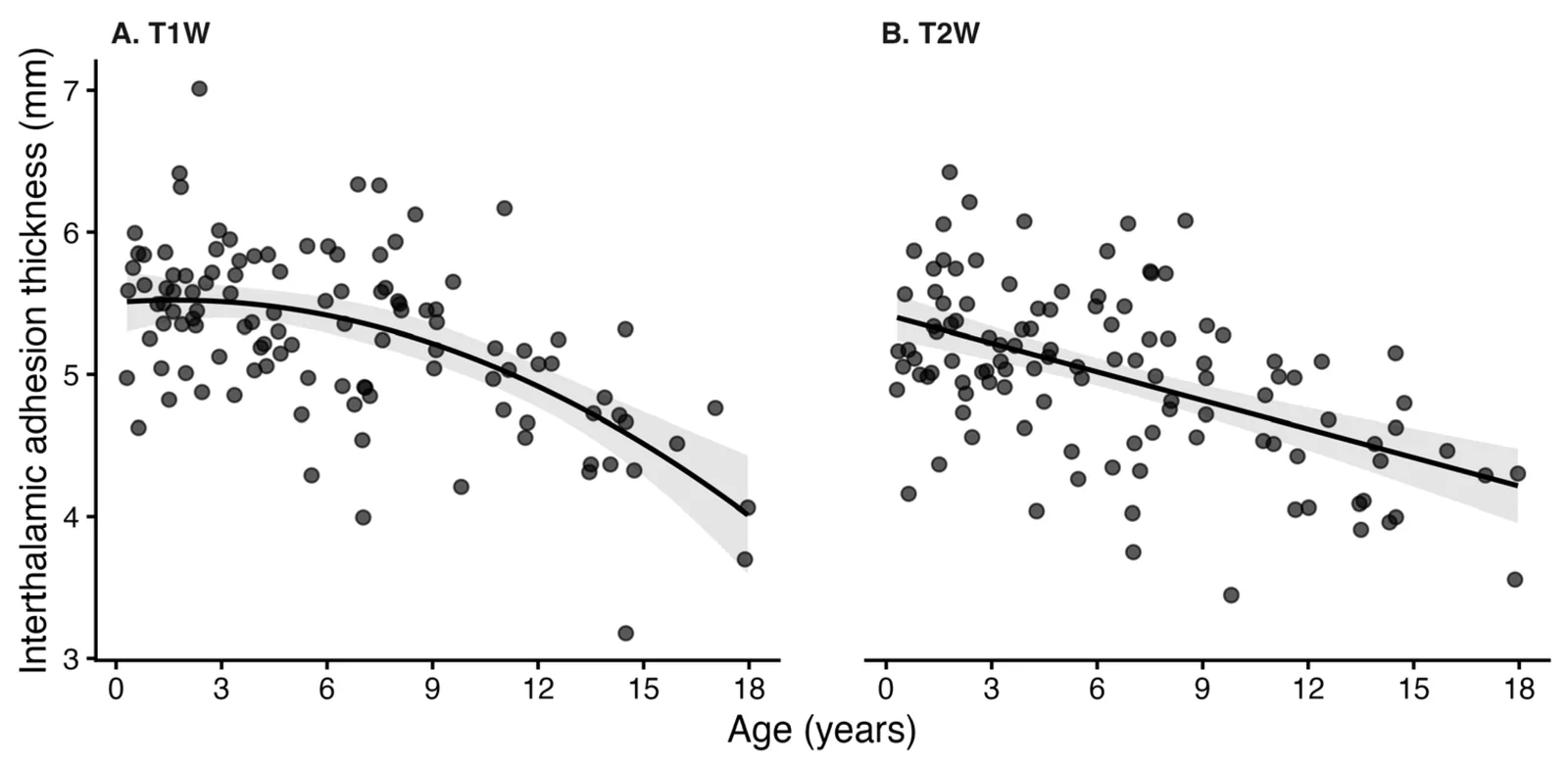
Association between age and interthalamic adhesion thickness (IAT) on (**A**) T1W and (**B**) T2W images. Each point represents the mean of the two observers’ measurements for one cat. Solid lines show the fitted quadratic model for T1W and the linear model for T2W; shaded areas indicate 95% confidence intervals for the estimated mean IAT. Models are unadjusted for brain height.

Univariable linear regression identified a significant negative association between age and IAT on T1W images (B = −0.071 mm/year, 95% CI −0.091 to −0.050, *p* < 0.001; R^2^ = 0.30) and on T2W images (B = −0.067 mm/year, 95% CI −0.088 to −0.046, *p* < 0.001; R^2^ = 0.27) (Figure 2).

After adjustment for brain height, brain width, sex/neuter status, breed group and scanner, age remained negatively associated with IAT on T1W images (B = −0.074 mm/year, 95% CI −0.094 to −0.053, *p* < 0.001) and T2W images (B = −0.067 mm/year, 95% CI −0.087 to −0.046, *p* < 0.001). Both models included 112 cats.

Brain width was positively associated with IAT on T1W (B = 0.055 mm/mm, 95% CI 0.011 to 0.099, *p* = 0.014) and T2W images (B = 0.072 mm/mm, 95% CI 0.027 to 0.117, *p* = 0.002). Brain height was positively associated with IAT on T2W images (B = 0.067 mm/mm, 95% CI 0.021 to 0.113, *p* = 0.005), but its coefficient was not statistically significant on T1W images (*p* = 0.086).

Brachycephalic cats had lower adjusted IAT on T1W (B = −0.818 mm, 95% CI −1.288 to −0.349, *p* < 0.001) and T2W images (B = −0.567 mm, 95% CI −1.046 to −0.089, *p* = 0.021). The 1.5 T scanner was associated with higher IAT than the 1.0 T scanner on T1W images (B = 0.222 mm, 95% CI 0.015 to 0.429, *p* = 0.035), but not significantly on T2W images (*p* = 0.922). None of the individual sex/neuter-status comparisons with castrated males was statistically significant on either sequence. Adjusted GVIFs ranged from 1.03 to 1.14, indicating no substantial collinearity.

On T1W images, a quadratic model fitted the data better than a linear model (ΔAIC = 4.9; F-test, *p* = 0.010), with a negative quadratic term (B = −0.0057, 95% CI −0.0100 to −0.0014), suggesting a nonlinear association between age and IAT.

On T2W images, the quadratic term did not improve model fit (ΔAIC = 0.2; *p* = 0.14), and a linear model was retained.

After the exclusion of kittens, the univariable linear association between age and IAT remained significant on T1W (B = −0.076 mm/year, 95% CI −0.098 to −0.054, *p* < 0.001; R^2^ = 0.31) and on T2W images (B = −0.075 mm/year, 95% CI −0.097 to −0.052, *p* < 0.001; R^2^ = 0.30). The T1W quadratic term remained significant (B = −0.0056, 95% CI −0.0104 to −0.0007, *p* = 0.025), but not the T2W quadratic term (*p* = 0.46).

After the exclusion of the five brachycephalic cats, the univariable linear association between age and IAT remained significant on both T1W images (B = −0.061 mm/year, 95% CI −0.082 to −0.040, *p* < 0.001; R^2^ = 0.25) and T2W images (B = −0.062 mm/year, 95% CI −0.084 to −0.040, *p* < 0.001; R^2^ = 0.23).

### 3.4. Comparisons Among Age Categories

**Figure 3:**
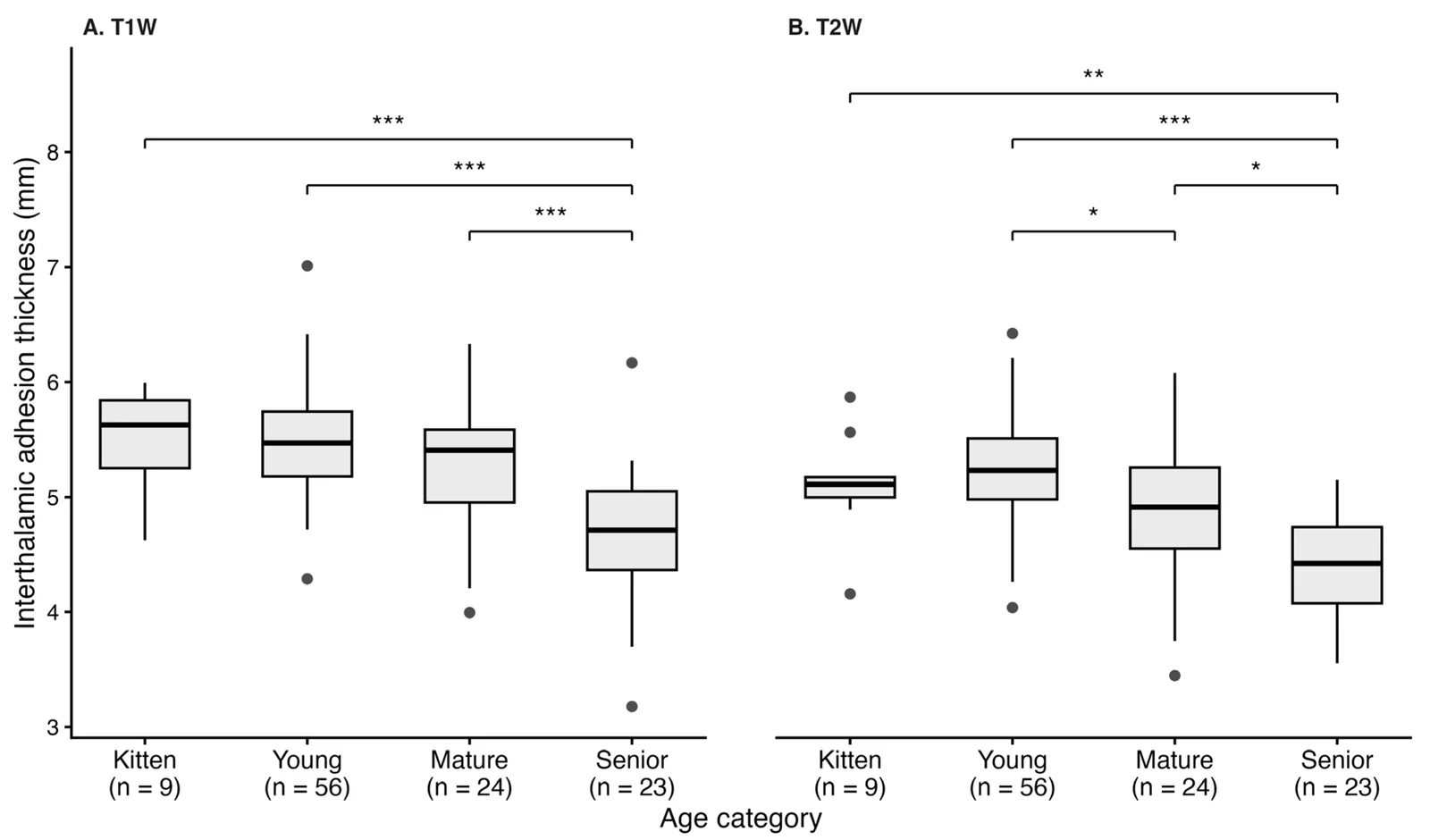
Interthalamic adhesion thickness (IAT) by age category on (**A**) T1W and (**B**) T2W images. Boxes show the interquartile range, with the median indicated by the central line. Whiskers extend to the most extreme observations within 1.5 times the interquartile range from the box; observations beyond the whiskers are shown as individual points. Sample sizes are indicated below each category. Brackets indicate significant pairwise comparisons with Tukey-adjusted *p* values: * *p* < 0.05, ** *p* < 0.01, and *** *p* < 0.001.

Results are summarized in Table 2 and Figure 3. For the T1W analysis, mean IAT values were 5.50 mm (4.62–5.99), 5.48 (4.29–7.01), 5.28 (3.99–6.33), and 4.68 (3.18–6.17) in the kitten (<1 year), young (1 to <7 years), mature (7 to <11 years), and senior (≥11 years) groups, respectively. A one-way ANOVA identified a significant difference in IAT among age groups on T1W images (*p* < 0.001). IAT was significantly lower in the senior group than in the young (mean difference −0.80 mm, 95% CI −1.14 to −0.47, *p* < 0.001), mature (−0.61 mm, 95% CI −1.00 to −0.21, *p* < 0.001) and kitten groups (−0.82 mm, 95% CI −1.35 to −0.29, *p* < 0.001). No significant differences were identified between the mature and young groups (−0.20 mm, 95% CI −0.53 to 0.13, *p* = 0.407). The kitten group did not differ significantly from the young (*p* = 1.000) or from the mature groups (*p* = 0.707).

For the T2W analysis, mean IAT values were 5.11 (4.16–5.87), 5.24 (4.04–6.42), 4.89 (3.45–6.08), and 4.43 (3.55–5.15) in the kitten (<1 year), young (1 to <7 years), mature (7 to <11 years), and senior (≥11 years) groups, respectively. A one-way ANOVA identified a significant difference in IAT among age groups on T2W images (*p* < 0.001). IAT was significantly lower in the senior group than in the young (mean difference −0.81 mm, 95% CI −1.14 to −0.47, *p* < 0.001), mature (−0.45 mm, 95% CI −0.85 to −0.06, *p* = 0.017) and kitten groups (−0.67 mm, 95% CI −1.20 to −0.14, *p* = 0.007). Mature cats also had a lower IAT than young cats (−0.35 mm, 95% CI −0.68 to −0.02, *p* = 0.030). The kitten group did not differ significantly from the young (*p* = 0.889) or from the mature group (*p* = 0.697).

## 4. Discussion

This study further characterizes age-related variation in IAT across defined feline life stages in a larger retrospective clinical MRI cohort of cats without apparent cognitive dysfunction than previously reported.

Our results identified a negative association between age, modeled as a continuous variable, and IAT on both T1W and T2W images. The negative association between age and IAT persisted on both sequences after adjustment for brain height, brain width, sex/neuter status, breed group, and scanner. In the secondary comparisons among life stages, senior cats had lower IAT than kittens, young adults and mature adults on both sequences. Mature adults also had lower IAT than young adults on T2W images, whereas this comparison was not statistically significant on T1W images. No statistically significant differences were identified between kittens and either young or mature adults.

These findings are consistent with those of two previous studies: one reported significantly lower mean IAT in cats aged 12 years or older compared with cats aged 1 to 6 years [10], and the other reported a significant decrease in interthalamic adhesion area and in the ratio of IAT to brain height with increasing age [11]. However, both studies included smaller samples. In contrast, our results differ from those of Hamamoto et al., who did not identify a significant decrease in IAT with age [5]. This discrepancy may be attributable to methodological differences, including the use of voxel-based morphometry and a smaller sample size (65 cats aged 1 to 16 years). Voxel-based morphometry may have limited sensitivity for detecting subtle changes in the interthalamic adhesion, a small midline structure, because spatial normalization, tissue segmentation, and smoothing may reduce anatomic specificity and obscure small focal differences.

In the present study, age was modeled as a continuous variable in the primary analyses, while categories adapted from the AAHA/AAFP Feline Life Stage Guidelines [12] were retained for secondary comparisons and clinical interpretation. Subdividing the senior category into cats younger than 15 years and those aged 15 years or older would have been of interest, given previous reports suggesting that age-related cerebral changes and/or behavioral changes attributed to FCDS become more pronounced in cats older than 15 years [3,10,13]. However, only four cats were aged 15 years or older, which was considered insufficient to support reliable comparisons between these subgroups. These cats were therefore retained within a single senior category.

The addition of a negative quadratic age term improved model fit on T1W images, suggesting a steeper reduction in IAT at older ages. This finding persisted after the exclusion of kittens. However, the quadratic term was not statistically significant on T2W images, and the small number of cats at the upper end of the age range limits the precision of the estimated curve in late life. Evidence for this curvature should therefore be considered exploratory.

Histopathological studies in aging cats have reported neuronal loss, synaptic degeneration, β-amyloid deposition and tau pathology [14,15,16,17]. Lower IAT may reflect neuronal loss within this structure and may also be associated with a reduced thalamic volume, as suggested by studies conducted in dogs [18] and humans [19]. Taken together, these observations suggest that IAT may be a relevant parameter for assessing age-related cerebral morphometric changes in cats.

The observed decrease in IAT with advancing age supports further investigation of IAT as a quantitative imaging marker of age-related cerebral changes in cats, as previously described in dogs [7]. Indeed, in healthy dogs, IAT has been reported to decrease with age [6,7,8]. Although the present study focused on cats without apparent cognitive dysfunction, it would be of interest to investigate whether brain atrophy is more pronounced in cats with FCDS, as has been suspected in cats [14] and demonstrated in dogs [7]. Given the challenges associated with diagnosing this condition, particularly because subtle behavioral changes may go unnoticed or be misinterpreted by owners, objective imaging measurements such as IAT may potentially contribute to a more objective assessment and facilitate earlier detection.

Several limitations should be considered when interpreting these findings. First, this retrospective study was conducted at a single referral hospital; therefore, the study population may not be representative of the general feline population. Cats underwent MRI for a range of clinical indications and had heterogeneous final diagnoses, which may have introduced selection bias despite the exclusion of cats with overt cerebral parenchymal lesions or owner-reported behavioral changes. Brachycephalic cats had lower adjusted IAT on both sequences, but only five were included, limiting the interpretation of this finding. The negative association between age and IAT remained significant after their exclusion.

Although the overall sample size was large, the distribution of cats across age groups was uneven. In the senior group, only four cats were aged 15 years or older, which limits the precision of estimates at the upper end of the age range.

Furthermore, cats were considered free of cognitive dysfunction based on retrospective medical records, without standardized behavioral assessment or a specific neurological examination. Subtle cognitive or behavioral changes may therefore have been present but unrecognized by owners.

In addition, MRI examinations were performed over an extended period with different scanners (1.0 Tesla until August 2015 and 1.5 Tesla thereafter), resulting in variation in image resolution. This technical heterogeneity may have affected the visualization of anatomic structures and the precision of IAT measurements, thereby contributing to measurement variability. Scanner was included as a covariate in the multivariable models and was associated with IAT on T1W but not T2W images. The negative association between age and IAT persisted after adjustment for scanner and the other covariates.

Finally, the use of a single dorsoventral measurement on 3 mm thick transverse images also introduces potential limitations related to partial-volume effects and slice positioning or orientation, which may affect the delineation of the interthalamic adhesion and measurement of its maximal thickness. The scanner-specific SEM and MDC95 estimates quantify the random component of interobserver measurement variability on the same images, but do not account for systematic errors shared by both observers or variability between separate MRI examinations. These estimates should therefore not be interpreted as validated thresholds for detecting longitudinal change in an individual cat. The present findings support a group-level association between age and IAT rather than individual-level diagnostic use. Midsagittal area measurements, three-dimensional volumetric measurements and approaches incorporating brain dimensions warrant evaluation as alternative or complementary morphometric methods. Their relative reproducibility and sensitivity to age-related changes should be assessed directly before superiority over a linear IAT measurement can be assumed.

## 5. Conclusions

Overall, IAT decreased with age in cats in this retrospective clinical MRI cohort. These findings support further evaluation of IAT as a simple morphometric marker of age-related cerebral changes in cats. Prospective studies involving cats with confirmed FCDS and age-matched control cats, standardized behavioral assessments and harmonized MRI protocols are required before clinical diagnostic use can be recommended.

## Figures and Tables

**Table 1:** Age (years), body weight (kg), sex, breed characteristics, brain width (mm) and brain height (mm) of sampled cats in the four age categories. BW: body weight; F: female; FS: spayed female; M: male; MC: castrated male; SD: standard deviation.

	Kitten [<1 y] (*n* = 9)	Young [1 to <7 y] (*n* = 56)	Mature [7 to <11 y] (*n* = 24)	Senior [≥11 y] (*n* = 23)
Median Age [min–max]	0.63 [0.31–0.96]	3.31 [1.18–6.88]	8.04 [7.00–10.78]	13.58 [11.02–17.96]
Mean BW [min–max]	2.7 [2.0–3.4]	4.4 [2.4–8.1]	5.2 [3.5–8.6]	4.2 [2.6–6.5]
Sex	F (2), M (3), FS (1), MC (3)	F (5), M (5), FS (18), MC (28)	F (1), M (0), FS (8), MC (15)	F (0), M (0), FS (13), MC (10)
Breed	European Shorthair (7), Maine Coon (1), Siamese (1)	European Shorthair (41), Birman (5), Maine Coon (3), Siamese (2), Chartreux (1), Exotic Shorthair (1), Norwegian Forest cat (1), Persian (1), Sphynx (1)	European Shorthair (21), Chartreux (1), Siamese (1), Persian (1)	European Shorthair (20), Siamese (1), Persian (2)
Mean brain width +/− SD	38.00 +/− 1.22	38.59 +/− 2.67	39.12 +/− 1.83	38.70 +/− 1.94
Mean brain height +/− SD	30.33 +/− 3.04	29.54 +/− 1.67	30.12 +/− 2.01	29.30 +/− 2.20

**Table 2:** Mean (min-max) and median (25th–75th percentiles) values for interthalamic adhesion thickness in the four different age categories on the T1W and T2W sequences.

	Kittens (*n* = 9)	Young (*n* = 56)	Mature (*n* = 24)	Senior (*n* = 23)
IAT T1W (mm)	Mean 5.50 (4.62–5.99) Median 5.63 (5.25–5.84)	Mean 5.48 (4.29–7.01) Median 5.47 (5.18–5.74)	Mean 5.28 (3.99–6.33) Median 5.41 (4.95–5.59)	Mean 4.68 (3.18–6.17) Median 4.71 (4.37–5.05)
IAT T2W (mm)	Mean 5.11 (4.16–5.87) Median 5.11 (5.00–5.17)	Mean 5.24 (4.04–6.42) Median 5.23 (4.98–5.51)	Mean 4.89 (3.45–6.08) Median 4.91 (4.55–5.26)	Mean 4.43 (3.55–5.15) Median 4.42 (4.08–4.74)

## Data Availability

The data presented in this study are available on request from the corresponding author due to confidentiality restrictions associated with retrospective clinical records.

## Abbreviations

The following abbreviations are used in this manuscript:

FCDS	Feline cognitive dysfunction syndrome
IAT	Interthalamic adhesion thickness
MRI	Magnetic resonance imaging
T1W	T1-weighted
T2W	T2-weighted
TR	Repetition time
TE	Echo time
FOV	Field of view

## References

[1] Sordo L., Gunn-Moore D.A. (2021). Cognitive dysfunction in cats: Update on neuropathological and behavioural changes plus clinical management. Vet. Rec..

[2] Landsberg G., Araujo J.A. (2005). Behavior Problems in Geriatric Pets. Vet. Clin. N. Am. Small Anim. Pract..

[3] Gunn-Moore D., Moffat K., Christie L.-A., Head E. (2007). Cognitive dysfunction and the neurobiology of ageing in cats. J. Small Anim. Pract..

[4] MacQuiddy B., Moreno J., Frank J., McGrath S. (2022). Survey of risk factors and frequency of clinical signs observed with feline cognitive dysfunction syndrome. J. Feline Med. Surg..

[5] Hamamoto Y., Yu Y., Asada R., Mizuno S., Hasegawa D. (2022). Age-related brain atrophy in cats without apparent neurological and behavioral signs using voxel-based morphometry. Front. Vet. Sci..

[6] Ji M., Kwon D., Kim S., Park H., Lee K., Yoon H. (2025). Interthalamic Adhesion Thickness and Its Ratio to Cerebral Height in Canine Brain MRI: Influence of Body Weight, MRI Sequence (T1W/T2W), and Imaging Plane. Vet. Radiol. Ultrasound.

[7] Noh D., Choi S., Choi H., Lee Y., Lee K. (2017). Evaluation of interthalamic adhesion size as an indicator of brain atrophy in dogs with and without cognitive dysfunction. Vet. Radiol. Ultrasound.

[8] Hasegawa D., Yayoshi N., Fujita Y., Fujita M., Orima H. (2005). Measurement of interthalamic adhesion thickness as a criteria for brain atrophy in dogs with and without cognitive dysfunction (dementia). Vet. Radiol. Ultrasound.

[9] Dewey C.W., Rishniw M., Johnson P.J., Davies E.S., Sackman J.J., O’Donnell M., Platt S., Robinson K. (2020). Interthalamic adhesion size in aging dogs with presumptive spontaneous brain microhemorrhages: A comparative retrospective MRI study of dogs with and without evidence of canine cognitive dysfunction. PeerJ.

[10] Babicsak V.R., Klein A.V., Tsunemi M.H., Vulcano L.C. (2018). Brain parenchymal changes during normal aging in domestic cats. Pesqui. Vet. Bras..

[11] Januel C., Morrow E., Gibson R., Gross A., de Sousa A.A., Rigby Dames B.A., Charvet C.J. (2026). Cat brains age like humans: Translating time shows pet cats live to be natural models for human aging. Biol. Open.

[12] Quimby J., Gowland S., Carney H.C., DePorter T., Plummer P., Westropp J. (2021). 2021 AAHA/AAFP Feline Life Stage Guidelines. J. Feline Med. Surg..

[13] Sordo L., Martini A.C., Houston E.F., Head E., Gunn-Moore D. (2021). Neuropathology of Aging in Cats and its Similarities to Human Alzheimer’s Disease. Front. Aging.

[14] Vite C.H., Head E. (2014). Aging in the Canine and Feline Brain. Vet. Clin. N. Am. Small Anim. Pract..

[15] Chambers J.K., Tokuda T., Uchida K., Ishii R., Tatebe H., Takahashi E., Tomiyama T., Une Y., Nakayama H. (2015). The domestic cat as a natural animal model of Alzheimer’s disease. Acta Neuropathol. Commun..

[16] Zhang C., Hua T., Zhu Z., Luo X. (2006). Age-related changes of structures in cerebellar cortex of cat. J. Biosci..

[17] Levine M.S., Adinolfi A.M., Fisher R.S., Hull C.D., Guthrie D., Buchwald N.A. (1988). Ultrastructural alterations in caudate nucleus in aged cats. Brain Res..

[18] Tapp P.D., Head K., Head E., Milgram N.W., Muggenburg B.A., Su M.Y. (2006). Application of an automated voxel-based morphometry technique to assess regional gray and white matter brain atrophy in a canine model of aging. NeuroImage.

[19] Walhovd K.B., Westlye L.T., Amlien I., Espeseth T., Reinvang I., Raz N., Agartz I., Salat D.H., Greve D.N., Fischl B. (2011). Consistent neuroanatomical age-related volume differences across multiple samples. Neurobiol. Aging.

